# Leveraging Machine Learning for Accurate Detection and Diagnosis of Melanoma and Nevi: An Interdisciplinary Study in Dermatology

**DOI:** 10.7759/cureus.44120

**Published:** 2023-08-25

**Authors:** Parsa Riazi Esfahani, Pasha Mazboudi, Akshay J Reddy, Victoria P Farasat, Monica E Guirgus, Nathaniel Tak, Mildred Min, Gordon H Arakji, Rakesh Patel

**Affiliations:** 1 Medicine, California University of Science and Medicine, Colton, USA; 2 Biology, Irvine Valley College, Irvine, USA; 3 Medicine, Midwestern University Arizona College of Osteopathic Medicine, Glendale, USA; 4 Dermatology, California Northstate University College of Medicine, Elk Grove, USA; 5 Health Sciences, California Northstate University, Rancho Cordova, USA; 6 Internal Medicine, East Tennessee State University Quillen College of Medicine, Johnson City, USA

**Keywords:** diagnostic accuracy, dermatologic imaging techniques, artificial intelligence (ai) in medicine, health public, deep neural network, melanoma skin cancer

## Abstract

This study explores the application of machine learning and deep learning algorithms to facilitate the accurate diagnosis of melanoma, a type of malignant skin cancer, and benign nevi. Leveraging a dataset of 793 dermatological images from the Kaggle online platform (Google LLC, Mountain View, California, United States), we developed a model that can accurately differentiate between these lesions based on their distinctive features. The dataset was divided into training (80%), validation (10%), and testing (10%) sets to optimize model performance and ensure its generalizability. Our findings demonstrate the potential of machine learning algorithms in enhancing the efficiency and accuracy of melanoma and nevi detection, with the developed model exhibiting robust performance metrics. Nonetheless, limitations exist due to the potential lack of comprehensive representation of melanoma and nevi cases in the dataset, and variations in image quality and acquisition methods, which may influence the model's performance in real-world clinical settings. Therefore, further research, validation studies, and integration into clinical practice are necessary to ensure the reliability and generalizability of these models. This study underscores the promise of artificial intelligence in advancing dermatologic diagnostics, aiming to improve patient outcomes by supporting early detection and treatment initiation for melanoma.

## Introduction

Melanoma, a type of skin cancer that originates from melanocytes is a global health concern [1]. It displays uncontrolled growth and the potential to spread if not detected and treated [2]. Interestingly, melanoma can develop from pigmented lesions called nevi on the skin [3]. It is worth noting that melanoma is not limited to the skin and can also affect the eyes appearing as ocular or uveal melanoma [4]. This particular type of cancer emerges in melanocytes within the uvea, which encompasses the iris, ciliary body, and choroid [5]. Although ocular melanoma is less prevalent than cutaneous melanoma, it poses a risk due to its tendency to metastasize to the liver [6]. The timely identification of accurate differentiation between benign and malignant lesions plays a crucial role in effectively diagnosing and managing melanoma [7].

Skin cancer, including melanoma, has long been associated with a burden of morbidity and mortality even prior to the coronavirus disease 2019 (COVID-19) pandemic [8]. Various imaging techniques have proven to be valuable tools for diagnosing and assessing melanoma and nevi. The non-invasive imaging method of dermoscopy allows for the visualization of subsurface structures and patterns within skin lesions [9]. Within the field of melanoma, various techniques such as slit lamp biomicroscopy, fundus photography, fluorescein angiography, and ultrasonography are utilized [10]. These diagnostic tools for skin and ocular melanomas provide clinicians with information to enhance diagnostic precision and make well-informed management decisions [11].

Emerging technologies like machine learning and deep learning algorithms offer possibilities for improving the diagnosis of melanoma and nevi. By employing artificial intelligence (AI) techniques to analyze imaging data, these algorithms can learn features and patterns that enable accurate differentiation between benign and malignant lesions [12]. The implementation of AI-based approaches holds the potential to enhance accuracy and improve decision-making for melanoma management. Such models have the potential to facilitate detection enable initiation of appropriate treatment and contribute to effective strategies in managing melanoma [13]. The integration of AI-based detection models for melanoma and nevi into workflows can enhance capabilities, particularly in healthcare settings where access to specialized dermatologists or sufficient resources may be limited [14].

The primary objective of this study is to develop a detection model that utilizes imaging data for the identification of melanoma and nevi. Our aim is to create a reliable model that can advance diagnosis by providing dermatologists with an objective and standardized tool [15]. Our research delves into the possibilities offered by machine learning algorithms (MLA) to enhance the diagnosis of melanoma and nevi through the analysis of imaging data. We aim to, with the aid of AI technology, create a detection model that's both precise and swift thereby making contributions to the worldwide endeavor against melanoma. By addressing the challenges in this explored realm of melanoma research, we hope to improve patient outcomes and foster advancements in this crucial field.

## Materials and methods

**Figure 1:**
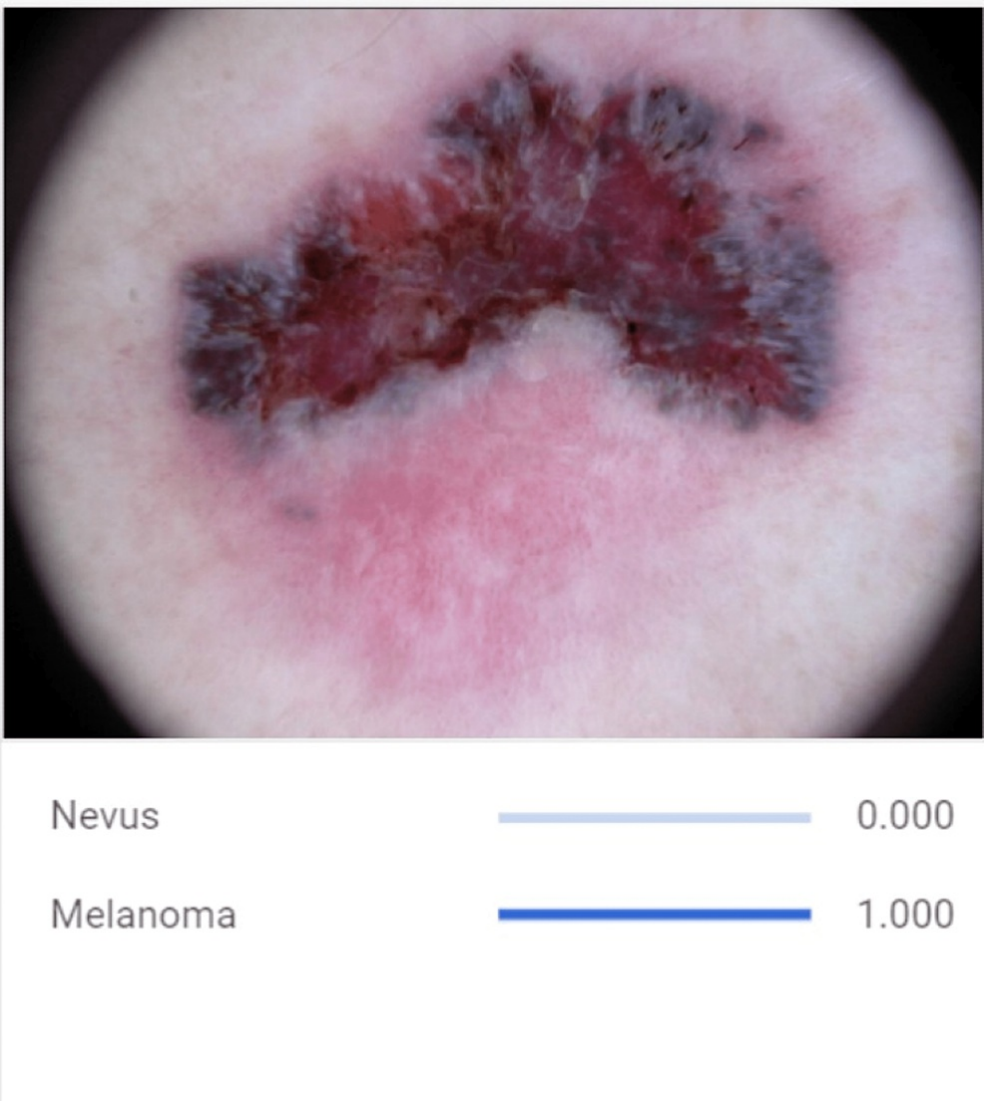
CNN Model Detecting Dermatoscopic Image of Melanoma Lesion The image showcases characteristic features of melanoma, including irregular borders, variegated colors, and asymmetry, which are used by the developed AI model for detection and diagnosis. CNN: convolutional neural network; AI: artificial intelligence

**Figure 2:**
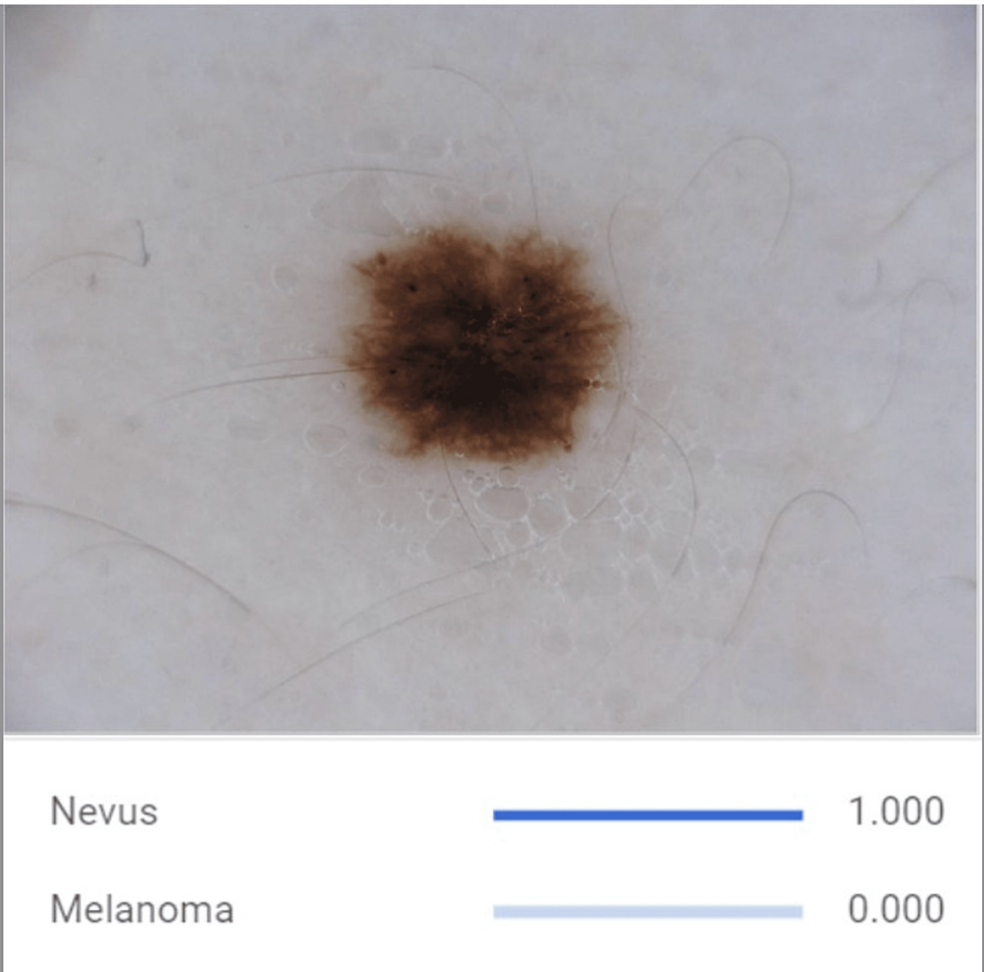
Developed CNN Model Detecting a Nevus The image highlights characteristic features of nevi, including regular borders, uniform coloration, and symmetry, which the developed AI model uses to distinguish these benign lesions from malignant melanoma. CNN: convolutional neural network; AI: artificial intelligence

This study aimed to develop a melanoma and nevus detection model using a dataset of 793 skin images obtained from the Kaggle online platform (Google LLC, Mountain View, California, United States). The dataset comprised 437 malignant melanoma (Figure 1) and 357 benign nevi (Figure 2) images. Machine learning techniques like convolutional neural networks (CNNs) were utilized to capture pertinent attributes and patterns from the dermatoscopy images. A CNN is a deep-learning model designed for image analysis. It utilizes layers of specialized filters to automatically learn and extract hierarchical features from input images, enabling it to recognize patterns, objects, and structures, while subsequent fully connected layers process these features for classification or regression tasks. The goal was to leverage Google Cloud’s collaborative platform (Google LLC) to create a CNN model to correctly identify distinctive features and patterns associated with melanoma, enabling accurate differentiation between malignant and benign skin lesions.

The dataset used for training was divided into three categories: (i) Training, (ii) Validation, and (ii) Testing. The first two categories (training and validation) were used to train the model, whereas the third category (testing) was used to test the model's efficacy. Of the total images, 80% were used for training, 10% for validation, and the remaining 10% for testing. Skin images were carefully selected from diverse sources to ensure a representative sample of melanoma and nevi cases. Kaggle.com provided a reliable and accessible resource for obtaining the dataset. The division of the dataset into training, validation, and testing sets allowed for model training on a substantial portion of the data while ensuring unbiased evaluation and generalizability. The training set was used to optimize the model's performance, while the validation set served to fine-tune the model's parameters and avoid overfitting. The testing set provided an independent sample for the final model evaluation and performance assessment. The use of skin images in this study provided a visual representation of melanoma and nevi, allowing the model to learn and recognize specific patterns associated with malignancy. Including both melanoma and nevi images ensured a comprehensive dataset to train and effectively validate the detection model.

Ethical considerations

The current investigation was considered exempt from the need for Institutional Review Board approval due to its exclusive use of a publicly accessible dataset, excluding any direct engagement with human participants. The dataset used in this study was sourced from openly available repositories, guaranteeing the full protection of personal information in terms of anonymity and confidentiality.

## Results

This study aimed to develop a robust melanoma and nevus detection model by employing MLAs on the skin image dataset, and the utilization of Kaggle.com as a data source and the carefully partitioned dataset enabled the development and evaluation of an accurate and reliable model for melanoma diagnosis.

**Figure 3:**
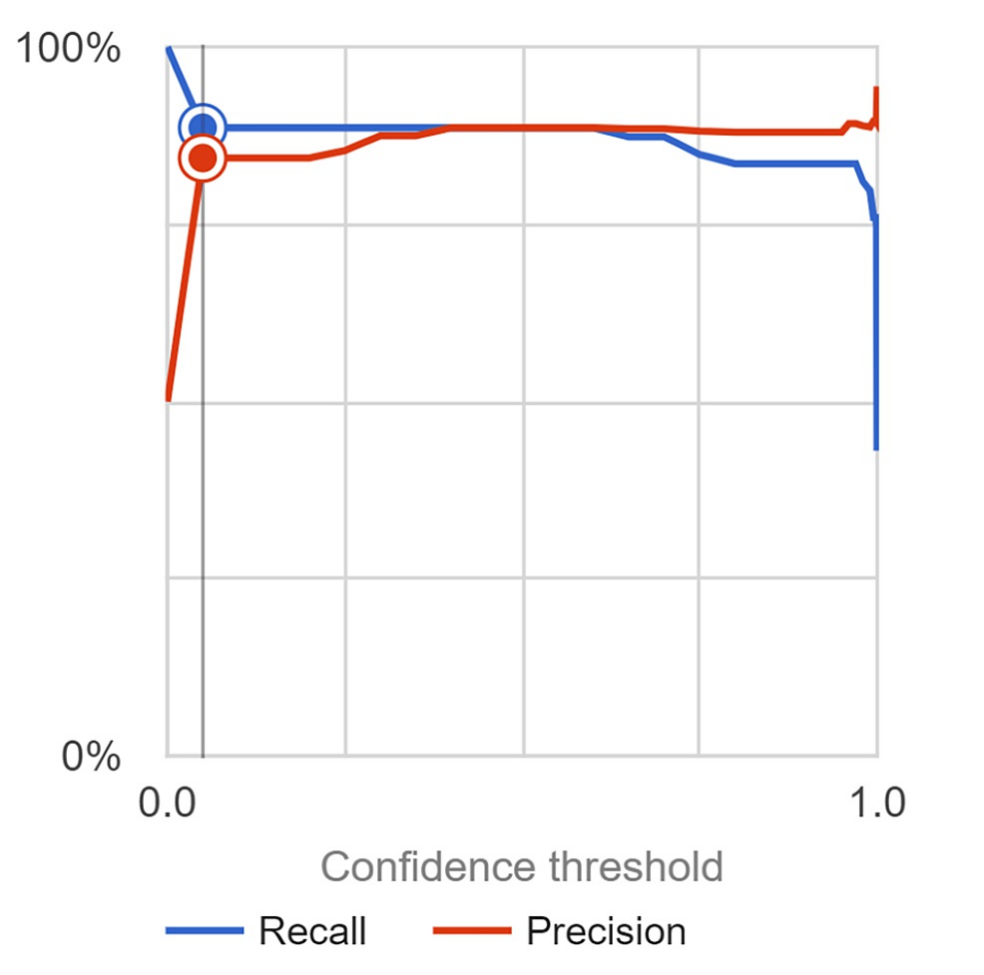
AUC Graph for Melanoma Detection Model Including Recall and Precision. This visual representation of area under the curve (AUC) graph showcases the precision and recall of the neural network model at varying confidence intervals. The researchers collected data for the model, employing a confidence interval of 0.05.

**Figure 4:**
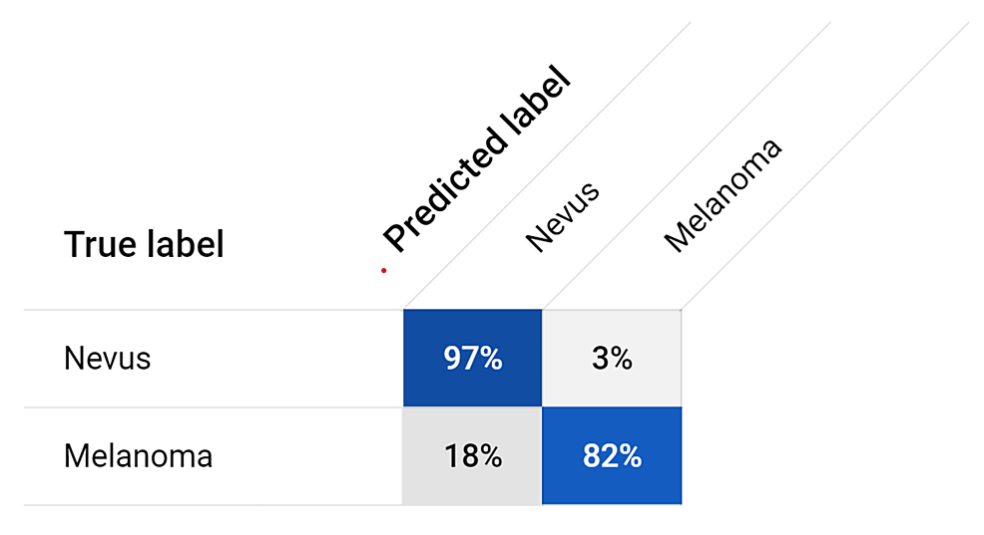
Confusion Matrix Different metrics of accuracy, precision, recall (sensitivity), specificity, and F-1 Score were calculated using the values from the confusion matrix.

The model achieved an average precision of 0.905, indicating its potential in accurately classifying melanoma and nevi cases. The precision value of 80.9% demonstrated the model's ability to correctly identify melanoma cases. The recall value, measuring the model's ability to capture all positive cases, was found to be 97.1%. Sensitivity, representing the true positive rate, was calculated as 82.02%, highlighting the model's effectiveness in correctly identifying melanoma cases. The specificity value, indicating the true negative rate, was determined to be 81.8%, showcasing the model's capability to accurately identify nevi cases. The F1 score, balancing precision and recall, was calculated as 0.883, further validating the model's overall performance, as depicted in Figure 3. The accuracy of the model, assessing its overall correctness in classifying melanoma and nevi cases, reached 88.6%. These values were calculated from the confusion matrix, as depicted in Figure 4. These results demonstrate the model's potential in accurately identifying melanoma cases while effectively distinguishing nevi cases. Further evaluation and refinement may be necessary to enhance the model's performance, but these findings highlight its ability to contribute to the field of melanoma diagnosis and management.

## Discussion

Melanoma, a type of skin cancer originating from melanocytes, poses a challenge in dermatology with regard to detection and diagnosis [16]. In this study, our objective was to explore the capabilities of machine learning and deep learning algorithms in detecting melanoma and nevi using imaging techniques. We utilized a dataset of images to develop a model that can effectively differentiate between malignant melanoma and benign nevi. The findings from our study highlight the potential of MLA in improving the accuracy and efficiency of melanoma and nevi detection. Dermatologic imaging techniques, including dermoscopy reflectance confocal microscopy, and optical coherence tomography are tools for visualizing and evaluating skin lesions. They provide dermatologists with information for enhancing accuracy and making informed decisions about the pathology of the skin lesion, whether it is cancerous, benign, or has the potential to become cancerous and metastasize [17]. These imaging methods offer high-resolution images of skin lesions that facilitate the identification of features and patterns associated with melanoma and nevi.

Our developed model exhibited performance metrics such as precision, recall, sensitivity, specificity, and accuracy. Its ability to accurately distinguish between melanoma and benign nevi demonstrates its clinical utility. By learning features and patterns indicative of melanoma MLAs contribute to the differentiation between malignant and benign skin lesions [18].

It is important to recognize the limitations of this study despite the results achieved by the developed model. The dataset utilized may not encompass the range of melanoma and nevi cases, which could hinder the model's ability to be applied in clinical scenarios. The model's performance might also be influenced by variations, image quality, and acquisition methods commonly encountered in practice [19]. To ensure the model's dependability and effectiveness, further validation studies incorporating larger datasets and validation using independent cohorts are necessary. These endeavors will enable assessing the model's performance across populations and imaging systems. Moreover, it is essential to consider the integration of the model into workflows evaluating its practicality and impact on care.

The potential of applying MLA to dermatologic imaging for melanoma and nevi detection is highly promising. The findings from this study contribute to efforts to enhance diagnosis and facilitate informed decision-making in dermatology. The presence of reliable models for melanoma and nevi detection can assist dermatologists in making precise diagnoses, ultimately leading to improved patient outcomes and more effective skin cancer management programs. This research study highlights the capabilities of machine learning and deep learning algorithms in creating a model, for detecting melanoma and nevi through dermatologic imaging. The findings underscore the significance of differentiating between non-cancerous skin lesions with imaging techniques playing a crucial role in aiding this procedure. Additional research, validation, and implementation, into practice, are essential to harness the benefits of these models and improve melanoma diagnosis and patient well-being.

## Conclusions

This study highlights the potential of MLA in accurately detecting and distinguishing between malignant melanoma and benign nevi using dermatologic imaging techniques. By leveraging the power of AI, this model provided standardized and objective analysis, which can aid dermatologists in making informed decisions and improving patient outcomes. This could be done by using AI as a screening technique in rural areas or areas lacking access to dermatologists allowing for a more effective and efficient healthcare system. However, further research and validation studies are needed to ensure the reliability and generalizability of these models in real-world clinical settings. Integrating machine learning-based melanoma and nevi detection into routine clinical practice holds great promise for enhancing diagnostic accuracy and optimizing patient care.

## References

[1] Davey MG, Miller N, McInerney NM (2021). A review of epidemiology and cancer biology of malignant melanoma. Cureus.

[2] Sarkar S, Horn G, Moulton K, Oza A, Byler S, Kokolus S, Longacre M (2013). Cancer development, progression, and therapy: an epigenetic overview. Int J Mol Sci.

[3] Damsky WE, Bosenberg M (2017). Melanocytic nevi and melanoma: unraveling a complex relationship. Oncogene.

[4] Tarlan B, Kıratlı H (2016). Uveal melanoma: current trends in diagnosis and management. Turk J Ophthalmol.

[5] Jovanovic P, Mihajlovic M, Djordjevic-Jocic J, Vlajkovic S, Cekic S, Stefanovic V (2013). Ocular melanoma: an overview of the current status. Int J Clin Exp Pathol.

[6] Rodríguez A, Dueñas-Gonzalez A, Delgado-Pelayo S (2016). Clinical presentation and management of uveal melanoma. Mol Clin Oncol.

[7] Jones OT, Ranmuthu CK, Hall PN, Funston G, Walter FM (2020). Recognising skin cancer in primary care. Adv Ther.

[8] Arnold M, Singh D, Laversanne M (2022). Global burden of cutaneous melanoma in 2020 and projections to 2040. JAMA Dermatol.

[9] Wu X, Marchetti MA, Marghoob AA (2015). Dermoscopy: not just for dermatologists. Melanoma Manag.

[10] Singh P, Singh A (2012). Choroidal melanoma. Oman J Ophthalmol.

[11] Davis LE, Shalin SC, Tackett AJ (2019). Current state of melanoma diagnosis and treatment. Cancer Biol Ther.

[12] Nambisan AK, Maurya A, Lama N (2023). Improving automatic melanoma diagnosis using deep learning-based segmentation of irregular networks. Cancers (Basel).

[13] Melarkode N, Srinivasan K, Qaisar SM, Plawiak P (2023). AI-powered diagnosis of skin cancer: a contemporary review, open challenges and future research directions. Cancers (Basel).

[14] Beltrami EJ, Brown AC, Salmon PJ, Leffell DJ, Ko JM, Grant-Kels JM (2022). Artificial intelligence in the detection of skin cancer. J Am Acad Dermatol.

[15] Alsaade FW, Aldhyani TH, Al-Adhaileh MH (2021). Developing a recognition system for diagnosing melanoma skin lesions using artificial intelligence algorithms. Comput Math Methods Med.

[16] Heistein JB, Acharya U, Mukkamalla SKR (2023). Malignant melanoma. StatPearls [Internet].

[17] Soglia S, Pérez-Anker J, Lobos Guede N, Giavedoni P, Puig S, Malvehy J (2022). Diagnostics using non-invasive technologies in dermatological oncology. Cancers (Basel).

[18] Das K, Cockerell CJ, Patil A, Pietkiewicz P, Giulini M, Grabbe S, Goldust M (2021). Machine learning and its application in skin cancer. Int J Environ Res Public Health.

[19] Combalia M, Codella N, Rotemberg V (2022). Validation of artificial intelligence prediction models for skin cancer diagnosis using dermoscopy images: the 2019 International Skin Imaging Collaboration Grand Challenge. Lancet Digit Health.

